# The novel protein kinase C epsilon isoform at the adult neuromuscular synapse: location, regulation by synaptic activity-dependent muscle contraction through TrkB signaling and coupling to ACh release

**DOI:** 10.1186/s13041-015-0098-x

**Published:** 2015-02-10

**Authors:** Teresa Obis, Núria Besalduch, Erica Hurtado, Laura Nadal, Manel M Santafe, Neus Garcia, Marta Tomàs, Mercedes Priego, Maria A Lanuza, Josep Tomàs

**Affiliations:** Unitat d’Histologia i Neurobiologia (UHN). Facultat de Medicina i Ciències de la Salut, Universitat Rovira i Virgili, Sant Llorenç 21, 43201 Reus, Spain

**Keywords:** PKC, PKC epsilon, Neuromuscular junction, Neurotransmission, Immunofluorescence, Electrical stimulation, Muscle contraction, TrkB

## Abstract

**Background:**

Protein kinase C (PKC) regulates a variety of neural functions, including neurotransmitter release. Although various PKC isoforms can be expressed at the synaptic sites and specific cell distribution may contribute to their functional diversity, little is known about the isoform-specific functions of PKCs in neuromuscular synapse. The present study is designed to examine the location of the novel isoform nPKCε at the neuromuscular junction (NMJ), their synaptic activity-related expression changes, its regulation by muscle contraction, and their possible involvement in acetylcholine release.

**Results:**

We use immunohistochemistry and confocal microscopy to demonstrate that the novel isoform nPKCε is exclusively located in the motor nerve terminals of the adult rat NMJ. We also report that electrical stimulation of synaptic inputs to the skeletal muscle significantly increased the amount of nPKCε isoform as well as its phosphorylated form in the synaptic membrane, and muscle contraction is necessary for these nPKCε expression changes. The results also demonstrate that synaptic activity-induced muscle contraction promotes changes in presynaptic nPKCε through the brain-derived neurotrophic factor (BDNF)-mediated tyrosine kinase receptor B (TrkB) signaling. Moreover, nPKCε activity results in phosphorylation of the substrate MARCKS involved in actin cytoskeleton remodeling and related with neurotransmission. Finally, blocking nPKCε with a nPKCε-specific translocation inhibitor peptide (εV1-2) strongly reduces phorbol ester-induced ACh release potentiation, which further indicates that nPKCε is involved in neurotransmission.

**Conclusions:**

Together, these results provide a mechanistic insight into how synaptic activity-induced muscle contraction could regulate the presynaptic action of the nPKCε isoform and suggest that muscle contraction is an important regulatory step in TrkB signaling at the NMJ.

## Background

Protein kinase C (PKC) comprises a family of serine-threonine protein kinases highly distributed in neural and neuromuscular tissues and involved in neurotransmitter release [1-5]. PKC isoforms, referred to as conventional, novel and atypical types, are activated by phosphatidylserine, diacylglycerol and/or Ca^2+^. Different isoforms exhibit distinct tissue distributions and different colocalizations of an activated PKC isoform with its endogenous protein substrates contributes to the functional diversity of the PKC isoforms [6,7]. Intracellular PKC-binding proteins known as RACKs (receptors for activated C-kinase) are essential to achieve the cellular specific patterns of distribution of an individual activated PKC isoform and therefore the functions of the PKC isoforms [8,9]. Therefore, the mechanisms that activate and compartmentalize PKC isoforms must be identified if the physiological functions of PKC are to be better understood.

Protein kinase C epsilon (nPKCε), a novel PKC isoform, is involved in the regulation of diverse cellular functions. It is highly expressed in the brain and several neural functions of nPKCε, including neurotransmitter release, have been identified [10]. It has been shown by Western blot analysis that nPKCε is also present in the skeletal muscle [11,12]. However, to date, no reports have been published on the localization and function of the nPKCε at the paradigmatic neuromuscular junction (NMJ). The present study is designed to examine the distribution of nPKCε at the NMJ of the adult rat and to know whether nPKCε level in synaptic membrane is modulated by synaptic activity and muscle contraction.

Myristolyated alanine rich C kinase substrate (MARCKS), a neuronal signal protein abundantly expressed in nerve endings, is an actin cross-linking protein that is highly phosphorylated on serine residues after PKC activation [13-15]. In addition, nPKCε regulates large dense-core vesicle release via phosphorylation of MARCKS [16]. MARCKS seems to be a key participant in actin cytoskeleton remodeling, which is the instrument to promote transfer of synaptic vesicles to the plasma membrane, relating thus MARCKS to neurotransmitter release [16-19]. Therefore, we studied if nPKCε activity results in phosphorylation of the substrate MARCKS at the NMJ. Finally, we investigated the possible involvement of nPKCε in ACh release.

We used immunohistochemistry and confocal microscopy to discriminate that nPKCε is exclusively located in the nerve terminals at the NMJ. We also disrupted the interaction between nPKCε and its specific RACK (and therefore its activation) with an isozyme-selective-translocation peptide inhibitor (εV1-2, [20]) in acute biochemical and electrophysiological experiments that also involved synaptic activity. We found that electrical activity-induced muscle contraction promotes changes in presynaptic nPKCε level through TrkB activity and that the nPKCε catalytic activity is related to the phosphorylation of MARCKS in an activity-dependent way. The results also demonstrate that nPKCε is involved in the PMA-induced ACh release mechanism at the NMJ.

## Results

### nPKCε and pnPKCε are expressed in adult and newborn skeletal muscle

**Figure 1:**
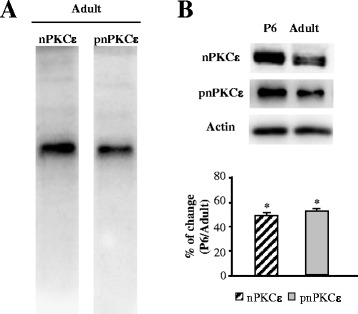
**nPKCε and pnPKCε are expressed in adult and newborn skeletal muscle. (A)** Western blot analysis of nPKCε and pnPKCε immunoreactivity in synaptic membrane in adult. **(B)** Western blot analysis of the novel PKC isoform ε was carried out to determine its presence in newborn (P6) and adult skeletal diaphragm muscle. Samples were also immunoblotted with an anti-phospho-PKCε antibody to identify specific phosphorylation of PKCε in resting conditions. The predicted molecular weight of the isoform is indicated. In all the experiments, we used actin as a loading control. The amount of protein loaded in each lane was 15 μg.

Western blot analysis using an antibody raised against the novel PKC isoform ε was carried out to determine the presence of the nPKCε isoform in young adult (P30-P40) diaphragm skeletal muscle. We also immunoblotted samples with an anti-phospho-PKCε antibody to identify the specific phosphorylation of nPKCε that is a requisite for PKC catalytic activity [21,22]. These experiments revealed significant amounts of this isoform (Figure 1A). The antibodies used only recognized the corresponding protein, reacting with a band consistent with its predicted molecular weight (manufacturer’s data sheets) (Figure 1A). No nPKCε presence was observed in brain excised tissues of KO mice in nPKCε, using this antibody [23]. Like other PKC isoenzymes, nPKCε must be primed through phosphorylation to display full enzymatic activity and respond to allosteric regulators.

We performed Western blot analysis and a quantitative study (VersaDoc, Bio Rad, Hercules, CA) to analyze the density of the band and evaluate the relative amount of the kinase isoform (and its phosphorylated form) in both the newborn (P6) and the adult. The results show that the expression of nPKCε isoform appears to be age-dependent being nPKCε more abundant in the newborn than in the adult (Figure 1B). Under resting conditions (isolated muscle does not receive action potentials from the motor neuron soma), we also found quite an abundant level of phosphorylated nPKCε and also pnPKCε is more abundant in the newborn than in the adult (Figure1B).

In conclusion, nPKCε is abundantly expressed and phosphorylated in the skeletal muscle. Subsequently, immunohistochemical analyses were performed to identify the cellular localization of the nPKCε isoform at the adult neuromuscular junction components (ie. muscle cells, Schwann cells or nerve terminals).

### nPKCε isoform is located in the nerve terminal at the neuromuscular junction

**Figure 2:**
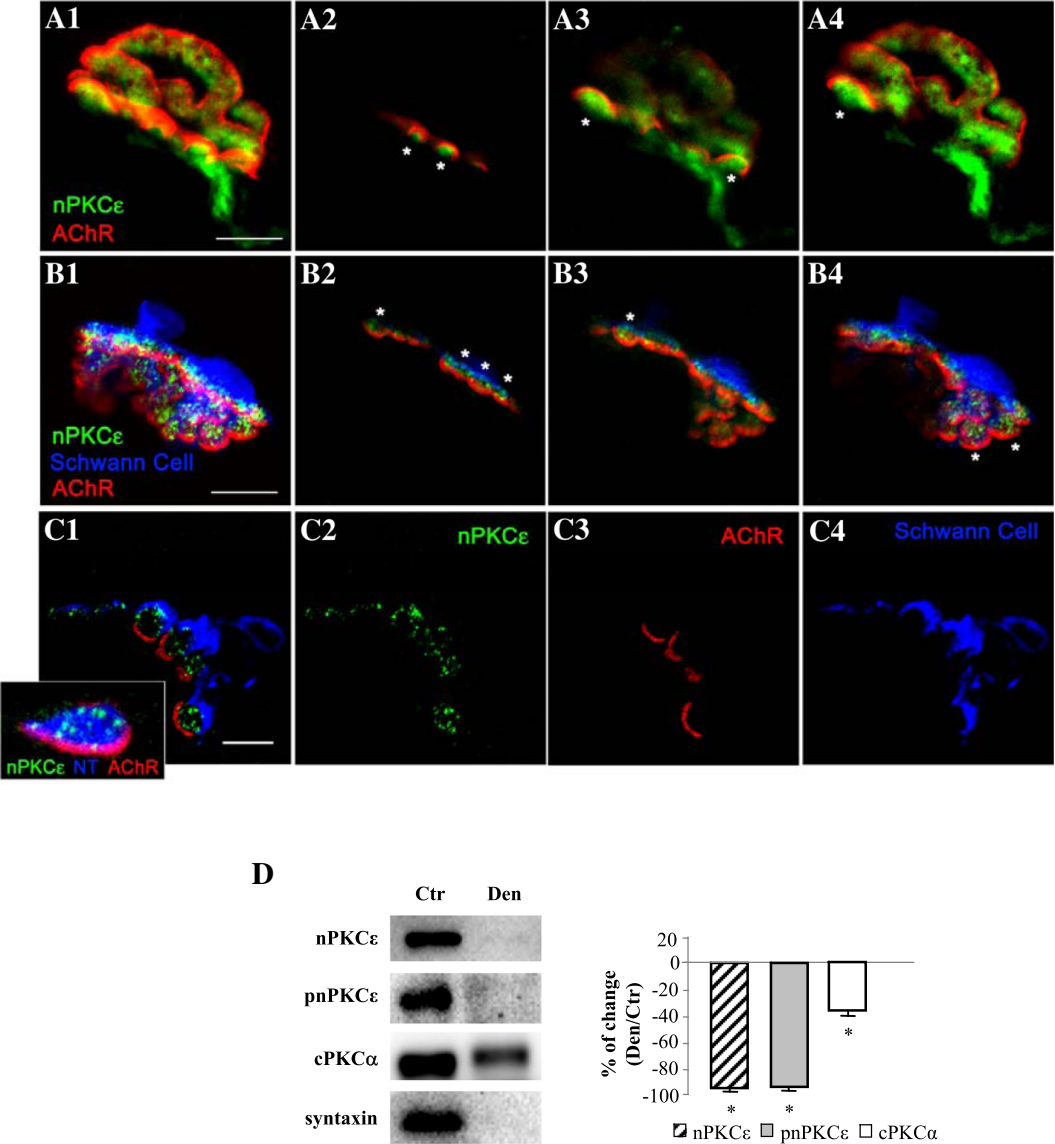
**nPKCε isoform is located in the nerve terminal at the adult neuromuscular junction. (A1)** shows a reconstruction of a NMJ stained for AChRs with fluorescent α-Bungarotoxin in red and immunolabeled with an antibody against nPKCε isoform in green. **(A2-A4)**, several semiconsecutive confocal optical sections from the same NMJ shown in A1. The (*) indicates nPKCε-positive nerve terminal buttons accommodated in the AChRs delimited synaptic gutters. **(B1)** shows a reconstruction of a NMJ triple labeled for AChRs (fluorescent α-BTX in red), the Schwann cell (immunolabeled with an anti S-100 antibody in blue) and nPKCε isoform in green. **(B2-B4)**, several confocal sections from the same NMJ shown in B1. The (*) also indicates nPKCε-positive granular immunolabeling concentrated on the nerve endings position over the synaptic gutters. The glia and muscle cell are not labeled. **(C)**, Immunohistochemistry in semithin sections from a whole-mount multiple-immunofluorescent stained muscle. In the colocalization picture **(C1)**, the triple staining that labeled nPKCε in green **(C2)**, AChRs in red **(C3)** and Schwann cells in blue, **(C4)** shows the nPKCε immunolabel only in the middle of the sandwich formed by glia and muscle cell. In the inset **(C1)**, the control triple staining that labeled PKCε (in green), AChRs (in red) and the nerve terminal (NT, in blue, neurofilament-200/syntaxin) shows that the nPKCε and nerve terminal markers are well colocalized. The bars in **(A)**, **(B)** and **(C)** indicate 10 μm. The bar in the inset indicates 2.5 μm. The NMJ are from LAL muscles. **(D)** Western blot analysis of nPKCε, pnPKCε, cPKCα and syntaxin in denervated EDL muscle.

Immunofluorescence staining coupled with confocal microscopy analysis was performed to determine the presence and localization of this isoform at the adult NMJ. Precise knowledge of the cellular localization of the nPKCε in pre-, post- and/or perisynaptic elements is crucial for elucidating the function/s of this protein. Immunofluorescence experiments were performed in the diaphragm and LAL muscles and immunoreactivity for nPKCε was identical in both muscles. All pictures in Figure 2 show intense immunoreactivity for nPKCε in the synaptic area identified with AChR labeling. Figure 2A shows a NMJ with double labeling: AChRs in red and nPKCε in green. Figure 2B shows a NMJ with triple labeling: AChRs in red, the Schwann cell in blue and nPKCε in green. These figures show nPKCε-positive green immunolabeling concentrated at the presynaptic position over the red postsynaptic gutters (asterisks). This can be seen both in the semiconsecutive confocal sections (A2-4, B2-4) and in the maximum projection (A1, B1) from the NMJs. The glia and muscle cell do not seem to be labeled (Figure 2B). In Figure 2A, it appears that pre-terminal axon is also nPKCε-positive.

To improve the analysis of the cellular distribution of the kinase at the NMJ we obtained semithin cross-sections from whole-mount multiple-immunofluorescent stained muscles [24]. We performed a triple staining in which nPKCε isoform (in green in Figure 2C2) was colocalized with molecular markers of the three cellular elements in the NMJ: muscle cell (nAChR marked with fluorescently labeled α-bungarotoxin, in red, Figure 2C3), Schwann cell (S-100, in blue, Figure 2C4) and nerve terminal (syntaxin and neurofilament, in blue, inset in C1) (Figure 2C). When the nPKCε was co-stained with α-BTX and S-100 (C1, colocalization picture), the nPKCε immunofluorescence (in green) was shown as several small granular areas located over the postsynaptic line of the nAChRs (in red) and externally surrounded by the Schwann cell (in blue). This label corresponds to the axonal buttons of the nerve terminal because it is colocalized with the syntaxin/neurofilament stain (labeled in blue in the inset in C1, Figure 2C) and also with syntaxin alone (not shown). Thus, the results indicate that nPKCε has a unique location in the synapse: it is exclusively expressed in the nerve terminals at the NMJ.

Denervation of the extensor digitorum longus muscle (EDL) was performed to confirm that nPKCε is located in the nerve. We denervated the muscle, waited 1–3 days, and studied the level of nPKCε by Western blots. The results show that nPKCε protein almost completely disappear after 1 day of denervation (ratio denervated/control 0.11 ± 0.08, p < 0.05) indicating that the most part of nPKCε is located in the nerve (Figure 2D). By contrast, cPKCα, an isoform ubiquitously located [25], slightly decreased (Figure 2D). Syntaxin has been used as a control of denervation. The residual presence of nPKCε may be related with blood vessels [11].

### nPKCε expression is modulated by synaptic activity

**Figure 3:**
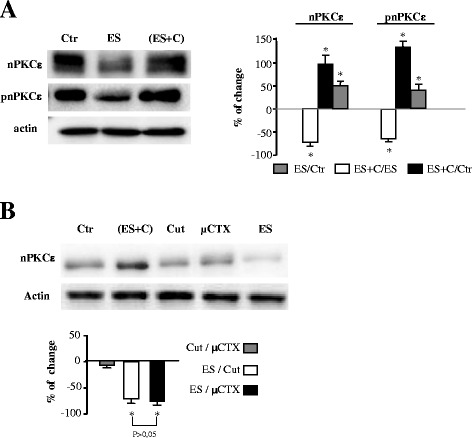
**nPKCε expression is modulated by synaptic activity. (A)** Western blot analysis of nPKCε and pnPKCε immunoreactivity levels in synaptic membrane in adult stimulated muscles with (ES + C) and without (ES) muscle contraction. **(B)** shows Western blots of nPKCε to compare the effect of the method to abolish muscle contraction (cut and μ-CgTx GIIIB (μCTX)). Quantitative data eliminate any possible artifact due to the method used to abolish muscle contraction. There are no significant differences between ratios μCTX vs Cut. The Western blot shows: (1) levels of nPKCε in non-stimulated muscles where no method was performed to abolish muscle contraction (Ctr), in cut muscles (Cut) and in muscles where muscle contraction was abolished by μ-CgTx GIIIB (μCTX); (2) nPKCε in electrically stimulated intact muscles where muscle contraction was abolished by μCgTx GIIIB (ES,μCTX); and (3) nPKCε in stimulated muscles resulting in contraction (ES + C). Experiments were performed using diaphragm muscle.

Synaptic function of PKC family has been shown to be regulated by activity [25], so we tested whether nPKCε synaptic level is modulated by synaptic activity. Therefore, we studied whether there was any change in the amount of nPKCε and its phosphorylated form in the synaptic membrane after electrical stimulation (with and without muscle contraction). Electrical stimulation of synaptic inputs to the diaphragm skeletal muscle (1 Hz for 30 minutes) and the resulting muscle contraction significantly increased the amount of nPKCε isoform and its phosphorylated form in the synaptic membrane (Figure 3A).

To separate the effect of the presynaptic stimulation (and synaptic transmission) from the effect of the muscle cell contraction, we performed experiments in which contractions were inhibited. When the contraction was inhibited by cutting the muscle fibers [26-28] or by using μ-CgTx-GIIIB [28,29], the electrical stimulation significantly decreased nPKCε and its phosphorylated form in the synaptic membrane (Figure 3A). Thus, nerve stimulation influences the kinase level in the synaptic membrane and this suggest that, with stimulation, the kinase could be detached from the membrane (and therefore stop its catalytic function), or alternatively stimulation entails some consumption of the enzyme. These results clearly indicate that muscle contractions induce an important change that reverses the effect of nerve stimulation by itself on nPKCε level in the synaptic membrane, suggesting that muscle contraction is necessary for nPKCε maintenance and its catalytic activity. Moreover, we performed several Western blots of nPKCε and pnPKCε to compare the effect of the method to abolish muscle contraction (cut and μ-CgTx GIIIB (μCTX)). Quantitative data show that (Figure 3B): (1) levels of nPKCε and pnPKCε are not significantly different in cut muscles and in muscles where muscle contraction was abolished by μ-CgTx GIIIB (μCTX); (2) nPKCε and pnPKCε significantly decreases in electrically stimulated intact muscles where muscle contraction was abolished by μCgTx GIIIB (ES/μCTX) in a similar way as occurs in electrically stimulated muscles where muscle contraction was abolished by cut (ES/Cut); (3) There are no significant differences between ratios μCTX vs Cut. These results eliminate any possible artifact due to the method used to abolish muscle contraction.

### Electrical activity-induced muscle contraction promotes changes in nPKCε through TrkB activity

**Figure 4:**
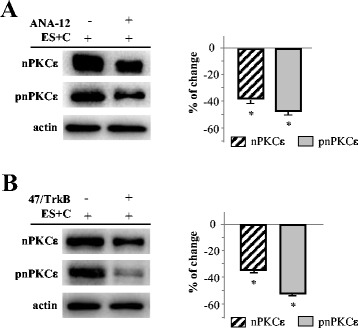
**Electrical activity-induced muscle contraction promotes changes in nPKCε isoform through trkB activity.** Western blot analysis of nPKCε and pnPKCε immunoreactivity levels in adult contracting muscles (ES + C) in the presence or not of the TrkB inhibitor Ana12 **(A)** or anti-TrkB antibody (47/TrkB) **(B)**. In all the muscles, the phrenic nerve was stimulated at 1 Hz for a 30-minute period and muscle contraction was not prevented. The quantitative histograms show the changes in (ES + C) with TrkB inhibitor with respect to (ES + C) and normalized with actin. The data are mean ± SD values from at least 5 independent experiments. *p < 0.05. Experiments were performed using diaphragm muscle.

The results set out above (Figure 3A) show that, in contracting muscles, the level of nPKCε are highly increased relative to the stimulated and non-contracting muscles, but also relative to the non-stimulated muscles. The results also show that when synaptic activity is not accompanied by muscle contractile activity, the level of nPKCε is decreased. Thus, we hypothesize that muscle contraction induces a change that can reverse the effect of nerve stimulation itself and a contraction-dependent neurotrophic support from postsynaptic site may make a contribution to this through the tyrosine kinase receptor B (TrkB). To demonstrate whether the activation of TrkB, as a result of muscle contraction, is critical to affect nPKCε’s level, we selectively suppressed TrkB activity in contracting diaphragm muscles using selective TrkB inhibitors. We used ANA-12 which showed direct and selective binding to TrkB and inhibits processes downstream of TrkB without altering TrkA and TrkC functions [30]. ANA-12 fully inhibits BDNF-induced TrkB phosphorylation and therefore prevents receptor activation. We also used an anti-TrkB antibody (47/TrkB) which effectively inhibits BDNF binding to TrkB receptors [31]. We measured resultant nPKCε and pnPKCε levels and found that TrkB blockade resulted in a significant decrease in the isoform (Figure 4). We obtained the same result with the two blockade methods. These results demonstrate that electrical activity-induced muscle contraction promotes increase in nPKCε and pnPKCε through TrkB activity suggesting that muscle contraction induce an increase of the interaction of a TrkB-specific neurothrophic factor with TrkB that enhance the intracellular signaling to increase the isoform levels in the synaptic membrane. Accordingly, exogenously added BDNF (10 nM, 30 minutes) in stimulated muscles without contraction significantly increased the amount of pnPKCε (58.78% ± 2.1% of change, p < 0.05). This result indicates that exogenous BDNF mimics muscle contraction effect on nPKCε levels. We choose the BDNF dose based in a previous dose–response and time-course study in the same muscle to determine their effect on the size of the evoked EPP [32].

### nPKCε regulates phosphorylation of MARCKS in skeletal muscle, in an activity-dependent way

To gain insight into the role of nPKCε we analyzed possible substrate phosphorylation. Although the translocation and phosphorylation of PKC is indicative of PKC activation [33], a more direct measure of the PKC activity is to detect PKC substrate phosphorylation in cells. Therefore, we wanted to know whether MARCKS phosphorylation is dependent on nPKCε at the NMJ and whether its phosphorylation depends on the synaptic activity (with and without muscle contraction).

**Figure 5:**
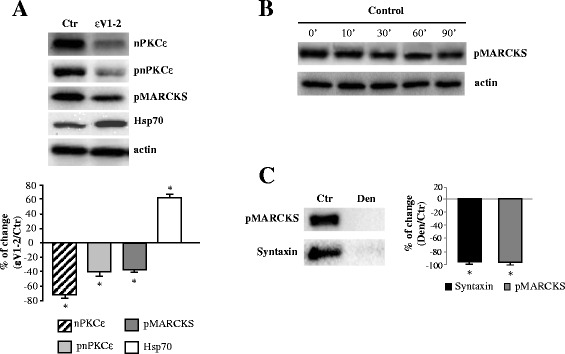
**nPKCε regulates phosphorylation of MARCKS in skeletal muscle. (A)** shows the Western blotting analysis of nPKCε, pnPKCε, pMARCKS and Hsp70 in control adult muscles (Ctr) and after incubation with peptide εV1-2 (10 min). **(B)** shows the time course of the level of pMARCKS in adult diaphragm muscle in resting conditions after muscle dissection. **(C)** shows the Western blotting analysis of pMARCKS and syntaxin in denervated EDL muscle. The quantitative histograms in A and C show the percentage of changes respect to the control and normalized with actin. The data are mean ± SD values from at least 5 independent experiments. *p < 0.05. Experiments were performed using diaphragm muscle.

We used the nPKCε-specific translocation inhibitor peptide, epsilonV1-2 (εV1-2; [20]), to block the isoform activity. We first wanted to show that the peptide εV1-2 inhibits the presence of nPKCε and pnPKCε in the synaptic membrane of the diaphragm muscle. Figure 5A shows that incubation with the peptide εV1-2 (100 μM) produces a rapid (10 min) and significant decrease in nPKCε and pnPKCε that is maintained after 60 minutes of incubation with the peptide (not shown). This decrease in the level of the nPKCε and pnPKCε induced by incubation with εV1-2 suggests that the nPKCε isoform may be tonically involved in some nerve terminal mechanism. No change in the level of the nPKCε and pnPKCε was observed in the presence of 100 μM of the scrambled peptide (not shown). Furthermore, the inhibition of nPKCε by the peptide has a different effect on nPKCε and pnPKCε (nPKCε decreases a 70% while pnPKCε a 40%). In this context, Figure 5A also shows that Hsp70 (heat shock protein 70), which has a role in prolonging the lifetime of activated PKC, significantly increases in the presence of the inhibitor peptide of nPKCε. This suggests a major interaction of Hsp70 with pnPKCε, which could prolong the lifetime of active nPKCε (pnPKCε) and sustain its function.

Next, we looked for pMARCKS expression in the skeletal muscle and in denervated muscle. Figure 5B shows the time course of the level of pMARCKS in diaphragm muscle in resting conditions indicating that skeletal muscle expresses pMARCKS and that pMARCKS is quite stable after muscle dissection. Denervation of EDL muscle totally removes pMARCKS (Figure 5C) indicating its presynaptic location. Note that syntaxin, a molecule exclusively located in the presynaptic component at the NMJ that has been used as a control, also disappears after denervation.

We next studied whether MARCKS phosphorylation is dependent on nPKCε in the skeletal muscle. Figure 5A shows that incubation for 10 minutes of the muscles with the peptide εV1-2 produces a significant decrease in pMARCKS indicating that nPKCε is related to the phosphorylation of MARCKS in the skeletal muscle.

**Figure 6:**
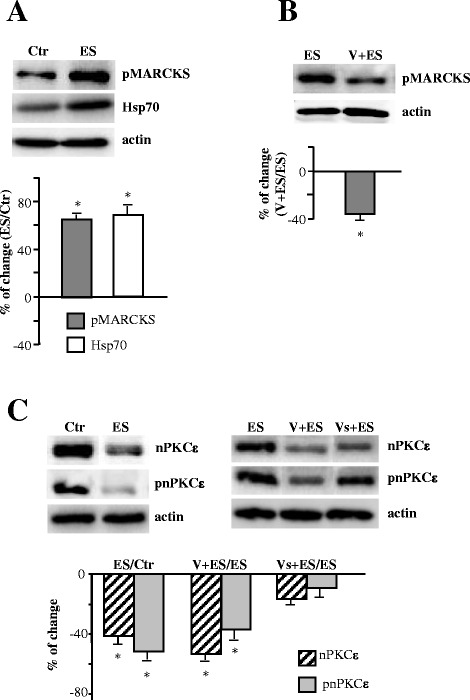
**nPKCε regulates phosphorylation of MARCKS in adult skeletal muscle, in an activity-dependent way. (A)** shows the Western blotting analysis of pMARCKS and Hsp70 in control (Ctr) and in stimulated muscles (ES). **(B)** shows the effects of εV1-2 incubation and electrical activity (V + ES) on the amount of pMARCKS. **(C)** shows the Western blotting analysis of nPKCε and pnPKCε in several conditions of stimulation: in control (Ctr), in stimulated muscles (ES), in stimulated muscles previously incubated with the blocking peptide (V + ES), in stimulated muscles previously incubated with the inactive peptide (Vs + ES). In all these experiments, the phrenic nerve was stimulated at 1 Hz for a 30-minute period and muscle contraction was prevented. The data are means ± SD values from at least 5 independent experiments and are normalized with actin. *p < 0.05. Experiments were performed using diaphragm muscle.

To determine whether stimulation (synaptic activity by itself, without muscle contraction) induces nPKCε phosphorylating activity on MARCKS in skeletal muscle, we subjected the protein extracts of stimulated muscles to phospho-MARCKS immunoblotting. WB analysis shows a significant increase in pMARCKS at 10 minutes indicating that phosphorylation of MARCKS depends on synaptic activity by itself (Figure 6A). In this condition, Hsp70 is also increased suggesting that pnPKCε could be stabilized by Hsp70 and therefore the kinase placed in the membrane could continue regulating the phosphorylation of MARCKS and increasing its amount. To further relate the effects of electrical activity and nPKCε activity we stimulated muscles previously incubated (100 μM, 30 minutes) with the blocking peptide (Figure 6B). We found a significant decrease in pMARCKS level, which emphasizes the role of nPKCε in MARCKS phosphorylation depending on the presynaptic activity. According, nPKCε and pnPKCε significantly decreased in these same conditions (Figure 6C) indicating also a possible additive effect and suggesting different levels in the modulation of nPKCε expression. Incubation with the inactive peptide (Vs) did not modify the level of the isoform obtained with electrical stimulation (Figure 6C).

**Figure 7:**
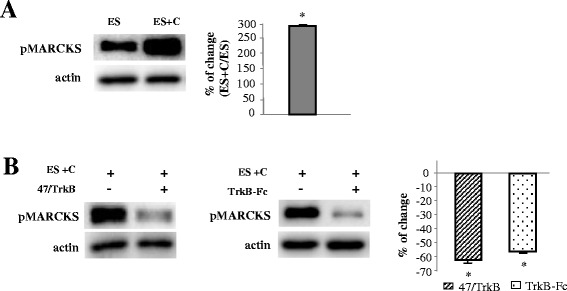
**Muscle contraction enhances phosphorylation of MARCKS through TrkB. (A)** shows the Western blotting analysis of pMARCKS in synaptic membrane in adult stimulated muscles with (ES + C) and without (ES) muscle contraction. **(B)** shows the Western blot analysis of pMARCKS immunoreactivity levels in adult contracting muscles (ES + C) in which has been selectively inhibited TrkB activity using the anti-TrkB antibody (47/TrkB) or the fusion protein trkB-Fc (to chelate endogenous BDNF/NT-4). In all these experiments, the phrenic nerve was stimulated at 1 Hz for a 30-minute period and muscle contraction was prevented (ES) or not (ES + C). The data are means ± SD values from at least 5 independent experiments and are normalized with actin. *p < 0.05. Experiments were performed using diaphragm muscle.

To test whether muscle contraction has an additional effect on MARCKS phosphorylation, we also analyzed pMARCKS level in electrical stimulated contracting muscles. Electrical stimulation accompanied by contraction (for 30 minutes) significantly increased the phosphorylation of MARCKS when compared with synaptic activity by itself (ES) (Figure 7A), indicating that synaptic activity with muscle contraction has a higher effect on the MARCKS phosphorylation than presynaptic activity by itself.

Because electrical activity-induced muscle contraction promotes changes in nPKCε through TrkB activity, next, we plan to know whether muscle contraction could affect MARCKS phosphorylation through TrkB activity. Thus, we analyzed the amount of pMARCKS in contracting muscles in which has been selectively inhibited TrkB activity using the anti-TrkB antibody (47/TrkB) or the fusion protein trkB-Fc (to chelate endogenous BDNF/NT-4). We found that TrkB blockade resulted in a significant decrease in pMARCKS (Figure 7B). We obtained the same result with the two blockade methods. These results demonstrate that electrical activity-induced muscle contraction promotes changes in pMARCKS through TrkB activity.

In summary, the data in this section indicates that nPKCε regulates phosphorylation of MARCKS in NMJ nerve terminal and that presynaptic activity enhances nPKCε-dependent MARCKS phosphorylation. Moreover, the synaptic activity-induced muscle contraction further enhances MARCKS phosphorylation through TrkB signaling.

### PMA-induced PKC-coupling to ACh release potentiation involves nPKCε

It is currently believed that PKCs can be strongly activated by such phorbol esters as phorbol 12-myristate 13-acetate (PMA), which potentiates ACh release. Therefore, although it has been reported that PMA has targets other than PKC for regulating neurotransmission [34,35], it should be determined whether the nPKCε plays a role in PMA-induced ACh release.

**Figure 8:**
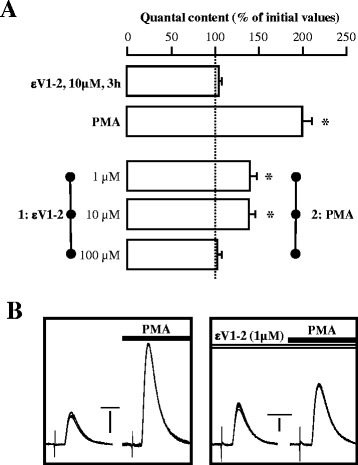
**PMA-induced PKC-coupling to ACh release potentiation involves nPKCε.** The histogram in **(A)** indicates that ACh release is increased by incubation with PMA. The dose-dependence analysis of the effect of the εV1-2 shows that the previous block of the nPKCε reduces (1 and 10 μM) and even abolishes (100 μM) the PMA potentiation of ACh release. For each kind of experiment: n = 5 adult muscles and a minimum of 15 fibers per muscle. *means p < 0.05. **(B)** shows the raw data of superimposed EPPs in the conditions described. Thus, the integrity of nPKCε seems a necessary condition if the PMA-induced transmitter release potentiation is to be fully expressed. Vertical bars: left, 10 mV; right, 5 mV. Horizontal bars: 4 ms. Experiments were performed using diaphragm muscle.

The histogram in Figure 8A shows that evoked ACh release can be strongly increased by incubation of the diaphragm muscle with PMA (raw data in Figure 8B left; see also [5,28,36]). Blocking the nPKCε isoform at low concentrations of the peptide εV1-2 (1 and 10 μM) significantly reduces the effect of PMA (~60%; raw data in Figure 8B right) whereas at high concentrations of εV1-2 (100 μM) PMA no longer potentiates ACh release. Data in Figure 8A are quantal content (the quantal content –M- was estimated with the direct method by recording mEPPs and EPPs simultaneously and then calculating the ratio: M = Average Peak EPP/Average Peak mEPP) and therefore reflects a presynaptic effect of εV1-2 on PMA-induced ACh release potentiation. To further discard any postsynaptic contribution we measured the average amplitude of the mEPPs after incubation with εV1-2 (1–100 μM) and PMA together or separately and found no difference above a non-significant 9% (P > 0.05) as compared with the non-incubated control. We conclude that nPKCε was activated by PMA and then coupled to potentiate release. Inhibition with the peptide εV1-2 eliminates the contribution of the epsilon isoform and, therefore, the PMA-induced PKC-coupling to ACh release potentiation significantly involves nPKCε activation.

These results suggest that nPKCε is involved in controlling transmitter release at the NMJ. It remains to further determine the specific mechanism of its contribution. However, bearing in mind all the results, we conclude that the nPKCε isoform is exclusively located in the presynaptic component of the NMJs and it is involved in the PMA-induced transmitter release mechanism at the NMJ. Moreover, synaptic activity-induced muscle contraction enhances nPKCε, and its catalytic function to increase phosphorylation of MARCKS, through TrkB activity.

## Discussion

Recent studies provide evidence that nPKCε could regulate distinct aspects of neural functions, including neurotransmitter release and signal transduction [10,37]. Here, we have examined the presence of the nPKCε in the adult NMJ, its synaptic activity-related expression, its regulation by muscle contraction and its involvement in ACh release.

### nPKCε expression and localization at the NMJ

It has been shown by Western blot analysis that nPKCε is widely distributed in the central nervous system [10] and skeletal muscle [11,12]. There is also a predominant presence of nPKCε mRNA in the brain [38,39]. Substantial amounts of nPKCε protein have been also detected in the rat brain, even at birth but a considerable increase was observed 1–2 weeks postnatal, which suggests that nPKCε is involved in constructing neural networks because it coincides with synapse formation [10]. Likewise, we found that substantial amounts of nPKCε were expressed in the rodent skeletal muscle being more abundant during the development than in the adult (roughly twice).

nPKCε and its phosphorylated form are located in the membrane fraction of the synapse-containing zone of the adult skeletal muscle. Furthermore (in correspondence with the denervation experiments), we localized nPKCε in the motor nerve terminals at the adult NMJ but not in the Schwann and postsynaptic muscle cells. Immunohistochemistry performed in the central nervous system reveals that the enzyme is most abundantly expressed in the hippocampus [10,40]. Interestingly, the immunoreactivity of the protein is evident in the nerve fibers and precise observation using electron microscopy reveals its presynaptic localization [6,40] as we found in the NMJ. These findings strongly indicate that nPKCε may be involved in presynaptic functions such as neurotransmitter release in mature synapses.

### Synaptic activity, nPKCε and pMARCKS

Because the synaptic function of PKC family is regulated by activity [25], we investigated whether synaptic activity regulates the translocation to the synaptic membrane of nPKCε and its phosphorylated form (which is an indication of its activation). Furthermore, because it has been reported that electrical stimulation-induced contraction increases the translocation of PKC to the membrane [25,41-43], we also wanted to study separately the effect on nPKCε of the presynaptic stimulation and synaptic transmission from the possible effect of the muscle cell contraction. Our results suggest that nPKCε has a role in some activity-related mechanism at the NMJ, and that muscle activity has an important impact on it. We found that electrical stimulation of synaptic inputs to the skeletal muscle and the resulting muscle contraction significantly increased the amount of nPKCε and its phosphorylated form in the synaptic membrane (Figure 3A). When the contraction was inhibited by cutting the muscle fibers [26-28] or by using μ-CgTx-GIIIB [28,29], electrical stimulation resulted in a significant decrease of nPKCε in the membrane (Figure 3). These results clearly indicate that muscle contraction induces an important signaling that reverses the effect of nerve stimulation by itself on nPKCε level in the synaptic membrane, suggesting that contraction is a necessary condition for the nPKCε catalytic function. A similar action of the muscle contraction related to cPKC isoforms has been previously described [25].

What could be the physiological significance of nPKCε levels being downregulated functionally by ES and, at the same time, being up-regulated by muscle contraction? The analysis of the substrate phosphorylation by nPKCε could help. Because there are no known nPKCε-specific substrates, we used MARCKS for several reasons. MARCKS is a widely distributed actin cross-linking protein that is highly phosphorylated on serine residues after PKC activation [13-15]. The phosphorylation of MARCKS has been used to assess both the activation and inhibition of PKC function [44]. MARCKS seems to be a key participant in actin cytoskeleton remodeling, which is the instrument to promote transfer of synaptic vesicles to the plasma membrane, relating thus MARCKS to neurotransmitter release [17]. In addition, nPKCε regulates large dense-core vesicle release via phosphorylation of MARCKS [16]. Our results show that MARCKS phosphorylation is dependent on nPKCε at the NMJ and its phosphorylation depends on the synaptic activity (with and without muscle contraction). Thus, the increase in the amount of the nPKCε and pnPKCε in the synaptic membrane after muscle contraction is in concordance with the increased phosphorylation of the PKC substrate MARCKS (Figure 7A). However, the results also show that nPKCε dependent-phosphorylation of MARCKS occurs when there is not muscle contraction (Figure 6A-B), indicating that presynaptic activity by itself is enough to induce some increase in pMARCKS. Nevertheless, pMARCKS is much higher in contracting muscles than in stimulated muscles without contraction (Figure 7A). Thus, it seems that the amount of pMARCKS depends on the amount of nPKCε active (which is higher when muscle contraction occurs) and, therefore, muscle contraction is necessary as a mechanism to increase the nPKCε level in the synaptic membrane. Interestingly, when nPKCε translocation is inhibited by εV1-2 in nerve-stimulated muscles, both nPKCε (Figure 6C) and pMARCKS (Figure 6B) significantly decrease indicating not only, as indicated above, that synaptic activity-induced phosphorylation of MARCKS depends on catalytic function of nPKCε, but also that activity-dependent nPKCε activation could results in its sustained work (may be to regulate phosphorylation of MARCKS) and subsequently in its detachment from the membrane that eventually could result in degradation or downregulation of the enzyme. In these conditions, Hsp70 (a heat shock protein which has a role in prolonging the lifetime of activated PKC) significantly increases (Figure 6A) suggesting a major interaction of Hsp70 with pnPKCε, which could prolong the lifetime of active nPKCε (pnPKCε) and sustain its function to promote phosphorylation of MARCKS. Together, these results indicated that synaptic activity by itself could activate nPKCε to enhance MARCKS phosphorylation at the NMJ and that muscle contraction could be necessary as a mechanism to increase the pnPKCε and pMARCKS levels. It has been described in other synapses and systems a link between phosphorylation of MARCKS and nPKCε [16,45,46]. However, because MARCKS can be phosphorylated by other PKCs we cannot fully discount that isoforms other than nPKCε phosphorylate this PKC-substrate in response to the increased activity, and related to the nPKCε activity, or even that other kinases are involved [47,48].

On the other hand, the blockade, in not stimulated muscles, of the translocation of nPKCε (and therefore its phosphorylating activity) with the εV1-2 peptide, decreased the level of nPKCε and pnPKCε (see Figure 5A). We interpret this result as the nPKCε activity inhibition as a result of the incubation with the specific translocation inhibitor peptide. This can be confirmed because the decrease in nPKCε is accompanied by a parallel decrease in pMARCKS (Figure 5A), which indicates not only that this isoform plays a role in phosphorylating MARCKS but also that the inhibitor peptide acts on the epsilon isoform.

### Effect of synaptic activity-induced contraction on nPKCε through TrkB activity

How is nPKCε level increased when muscle contraction is not prevented? Recently, several evidence support muscle activity-dependent BDNF and TrkB signaling as a key regulator of neuromuscular function. First, BDNF has been identified as a contraction-inducible protein in human skeletal muscle [49]. Secondly, basal levels of neuromuscular activity are required to maintain normal levels of BDNF in the neuromuscular system [50]. Finally, BDNF and TrkB contribute to the neuromuscular junction transmission [32,51]. Accordingly, we aimed to investigate whether nerve induced-muscle activity would produce a neurotrophin inducing a signaling pathway through TrkB to affect presynaptic nPKCε and its catalytic activity. Our results show that blockade of TrkB prevents muscle contraction-induced nPKCε increase (Figure 4A-B) and phosphorylation of MARCKS (Figure 7B) indicating that a neurotrophin acts through TrkB to increase nPKCε and pMARCKS on the nerve terminal. Moreover, we found that phosphorylation of nPKCε increases significantly when stimulated muscles were incubated with BDNF exogenous, indicating that BDNF could be the neurothrophin that activates TrkB to enhance nPKCε action and pMARCKS on the nerve terminal. It could be speculated that TrkB works to regulate nPKCε (and pnPKCε) by phosphorylating PLCγ (phosphoinositide-specific phospholipase C γ). Phosphorylation of TrkB on Tyr785 recruits PLCγ to the receptors, and the enzyme becomes activated upon tyrosine phosphorylation [52,53]. Activated PLCγ hydrolyses PI(4,5)P_2_ (phosphatidylinositol 4,5-biphosphate) to generate inositol tris-phosphate (IP_3_) and DAG, which activates nPKCε (once the isoform has been previously phosphorylated by PDK (3-phosphoinositide dependent protein kinase) and autophosphorylated [54-56]. These findings provide mechanistic insight into how synaptic activity induced-muscle contraction could regulate the presynaptic action of the nPKCε and suggest that muscle contraction is an important regulatory step in TrkB signaling at the NMJ.

### Effect of nPKCε on PMA-induced PKC-coupling to ACh release potentiation

One aim of the present study is to determine whether nPKCε is involved in the neurotransmission mechanism. Although it has been reported that PMA has targets other than PKC for affecting neurotransmission (Munc13) [34,35], PMA strongly activates PKC family to enhance neurotransmission [5,36,57-60]. Here, we show that blocking the nPKCε with εV1-2 fully inhibited (Figure 8) the PMA-induced pharmacologic potentiation of ACh release in a concentration-dependent way indicating that nPKCε plays a role in neurotransmission at the NMJ. Therefore, nPKCε has a key role regulating the ACh release by itself or by modulating the action of other PKCs isoforms. It has been demonstrated that PKC family is involved in the modulation of ACh release at the NMJ [60]. Moreover, exogenously added BDNF potentiates evoked ACh release in a TrkB receptor dependent manner [32]. Furthermore, the TrkB signaling needs an operative PKC pathway to couple to the release mechanism and potentiate it [61]. nPKCε could be related with this PKC-TrkB mechanism that modulates ACh release at the NMJ. Future experiments will be necessary to determine how nPKCε is critical to the maintenance of transmitter release and to better understand how muscle contraction-induced phosphorylation of MARCKS, regulated by nPKCε, is involved in neuromuscular transmission. Furthermore, nPKCε may work in parallel with the active zone positioning and priming protein Munc13 to enhance neurotransmission because recent studies proposed two probably converging pathways stimulated by phorbol esters (and DAG) to induce potentiation (PKC- and Munc13-dependent) [62,63]. It has been proposed that a PKC/Munc18-1 dependent-redistribution of synaptic vesicles after phorbol ester stimulation (via PKC phosphorylation of the SNARE/SM fusion protein Munc18-1), may be the morphological correlate of the increased release efficiency during potentiation [62]. In this redistribution, MARCKS may be involved because their role in actin cytoskeleton remodeling, which is the instrument to promote transfer of synaptic vesicles to the plasma membrane.

**Figure 9:**
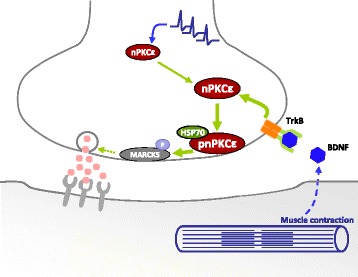
**Diagram of proposed mechanism mediating activity-dependent regulatory function of nPKCε on ACh release linked to muscle contraction.** The diagram illustrates how synaptic activity induced-muscle contraction could regulate the presynaptic action of the nPKCε through TrkB signalling in the neurotransmission release. The activity of the nPKCε isoform requires muscle cells to contract if the presynaptic membrane level of nPKCε is to be maintained or increased (probably with the involvement of the stabilizing action of HSP70). Finally, we hypothesize that nPKCε is functionally related in the neurotransmission mechanism on the NMJs.

In physiological conditions, nerve action is linked to muscle contractions, therefore it is important to provide a mechanism linking muscle contraction to nerve function. Figure 9 shows a diagram illustrating how synaptic activity-induced muscle contraction could regulate the presynaptic action of the nPKCε through TrkB signaling to enhance phosphorylation of MARCKS that could be related to the neurotransmitter release.

## Conclusion

In summary, our results indicate that nPKCε is exclusively located in the presynaptic component of the NMJs, is regulated by synaptic activity involving muscle contraction through TrkB function to enhance phosphorylation of MARCKS and finally, nPKCε is involved in ACh release.

## Material and methods

### Animals

Diaphragm and levator auris longus (LAL) muscles of young adult and postnatal male Sprague–Dawley rats (30–40 and 6 days; Criffa, Barcelona, Spain) were used to perform stimulation experiments, immunohistochemistry and electrophysiological experiments. Denervation experiments were performed in the extensor digitorum longus muscle (EDL). The animals were cared for in accordance with the guidelines of the European Community Council Directive for the humane treatment of laboratory animals.

### Materials

#### Antibodies

Primary antibodies used for Western blot and immunohistochemistry analysis were obtained from different sources, as follows: rabbit anti-PKCε, goat anti-phospho-PKCε (Ser729) and rabbit anti-PKCα polyclonal antibodies were purchased from Santa Cruz Biotechnology (Santa Cruz, CA); rabbit anti-PKCε and rabbit anti-phospho-PKCε (Ser729) polyclonal antibodies were obtained from Upstate Biotechnology (Millipore, Lake Place NY); mouse anti-PKCα monoclonal antibody was purchased from BD Transduction Laboratories (Lexington, KY); rabbit anti-phospho-MARCKS (myristoylated alanine-rich C kinase substrate) (S152/S156) polyclonal antibody from R&D systems (Biotecnology, Minneapolis); rabbit anti-HSP70/HSP72 polyclonal antibody from Enzo (Life Sciences, Inc.); goat anti-GAPDH from Imgenex (San Diego, CA) and rabbit anti-pan-actin polyclonal antibody from Cell Signaling Technology, Inc (Beverly, MA). The secondary antibodies used in the Western blot were goat anti-mouse and donkey anti-rabbit conjugated to HRP (Horseradish Peroxidase) from Jackson Immunoresearch and rabbit anti-goat HRP from Molecular Probes (Eugene, OR). For the immunohistochemistry we also used antibodies that are commonly used as markers to differentially detect the parts of the NMJ (syntaxin, neurofilament-200 and S100): the mouse monoclonal antibody and the rabbit polyclonal anti-syntaxin antibody (Sigma, St Louis, MO); the monoclonal and polyclonal anti-neurofilament-200 antibodies (Sigma, St Louis, MO); the rabbit anti-S100 antibody (Dako, Carpinteria, CA) and the mouse anti-S100 antibody (Acris, Germany). The secondary antibodies used were donkey anti-rabbit or anti-mouse conjugated to Alexa Fluor 488 and Alexa Fluor 647 from Molecular Probes (Eugene, OR). Postsynaptic acetylcholine receptors (AChRs) were detected with α-bungarotoxin (α-BTX) conjugated to tetramethyl rhodamine iso-thiocyanate (TRITC) from Molecular Probes (Eugene, OR).

As a control, the primary antibodies were omitted from some muscles during the immunohistochemical and Western blot procedures. These control muscles never exhibited positive staining or revealed bands of the appropriate molecular weight with the respective procedures. In double-staining protocols, omitting either one of the two primary antibodies completely abolished the corresponding staining and there was no cross-reaction with the other primary antibody. Pretreatment of a primary antibody with an excess of the appropriate blocking peptide (between three- and eightfold by weight) in skeletal muscle tissue prevented immunolabeling. All the primary antibodies used detect a single band with the referenced molecular weight on Western blot (manufacturer’s data sheets; see also Figure 1 for nPKCε and pnPKCε skeletal muscle).

#### Reagents

For the different treatments we used substances that modulate PKC activity: Phorbol 12-myristate 13-acetate (PMA, Sigma) was made up as 10 mM stock solution in dimethylsulfoxide (DMSO; Tocris, Ellisville, MO, USA). The working solution was PMA 10 nM. εV1-2, nPKCε-specific translocation inhibitor peptide (myristoylated PKC-ε V1-2 peptide, EAVSLKPT) from Calbiochem (Merk, Germany) was made up as 1 mM in distilled water or normal Ringer solution. Working solutions were 1, 10 and 100 μM. As a negative control of the nPKCε-specific translocation inhibitor peptide we used scrambled εV1-2 peptide (εV1-2-s, LSETKPAV), containing the same aminoacids as the inhibitor peptide but in a different sequence, from Calbiochem (Merk, Germany). Working solutions were 1, 10 and 100 μM.

#### TrkB receptor-inhibitors

The TrkB ligand N2-(2-{[(2-oxoazepan-3-yl)amino]carbonyl}phenyl)benzo[b]thiophene-2-carboxamide (ANA-12; cat. no. BTB06525; MW 407.5 g/mol) was purchased from Maybridge (Cornwall, UK) and was made up as a 10 mM in DMSO. Anti-TrkB (clone 47/TrkB; cat. no. 610102; 250 μg/ml) was obtained from BD Transduction Laboratories (Lexington, KY). Recombinant human trkB/Fc Chimera (trkB-Fc; 688-TK from R&D Systems, Minneapolis), 100 μg/ml. Working solutions: ANA-12, 10 μM; anti-TrkB, 10 μg/ml; trkB-Fc, 1–5 μg/ml.

### Stimulation of the muscle and incubations with εV_1–2_ peptide and PMA

In all the experimental protocols, the diaphragm muscle from young adult rats were excised together with the phrenic nerve and placed in oxygenated Ringer solution (see below) continuously bubbled with 95% O2/5% CO_2_ at room temperature. To stimulate the muscle, the phrenic nerve was stimulated at 1 Hz for a 10- or 30-minute period by an A-M Systems 2100 isolated pulse generator (A-M System, Carlsborg, WA). To study separately the effect of the presynaptic stimulation (and synaptic transmission) from the effect of the muscle cell contraction, we performed experiments in which contractions were prevented (ES) or not (ES + C). The muscle was prevented from contracting by cutting on either side of the main intramuscular nerve branch (see below). We also performed experiments to discard any possible artifact due to the muscle fiber cutting. Cut muscles were compared with muscles where muscle contraction was abolished by using μ-conotoxin GIIIB (μ-CgTx-GIIIB, 3 μM −1.5 μM, from ICS, International Clinical Service GmbH, München). The two methods used to prevent muscle contraction did not show differences with regard to electrophysiological parameters of the neurotransmission (see also later in Results).

We used a nonpharmacological tool to inhibit specifically nPKCε isoform. We used the nPKCε-specific translocation inhibitor peptide, epsilonV1-2 (εV1-2; [20]), to block the isoform activity so that we could analyze its possible constitutive involvement in cell functions in the resting and stimulated NMJs. This peptide interferes in the nPKCε interaction with the anchoring protein epsilon-RACK and, therefore, inhibits the anchoring of nPKCε near its substrates (and translocation to the membrane of the active isoform) and prevents any subsequent substrate phosphorylation and activity [64]. For nPKC, the PKC–RACK interaction occurs via a C2-like domain that does not bind Ca^2+^ [64,65]. Thus, the classic calcium-dependent cPKCs are not inhibited with the peptide εV1-2 because they do not possess this C2-like region. In addition, studies using peptide translocation inhibitors of nPKCε (εV1-2) and nPKCδ (δV1) demonstrated that these isoforms (nPKCε and nPKCδ) can have opposing actions within a given cell type (cardiomyocyte) [66-68] and also showed the isoform specificity of the peptides. There are several evidence that show that the effects found using the nPKCε-specific translocation inhibitor peptide can be confirmed when nPKCε knockout mice are used [69,70]. The effect of εV1-2 (100 μM) was studied in parallel to the effect of the scrambled version of this peptide (εV1-2-s, 100 μM). In some experiments, stimulated muscles were previously incubated with the peptide εV1-2 or with its inactive form (30 minutes).

To activate PKC family with PMA, a potent but non-selective-isoform PKC activator, the diaphragm muscles were incubated for 10, 30 or 60 minutes on a Sylgard-coated Petri dish containing Ringer solution for the control muscles, or εV1-2 (100 μM), scrambled εV1-2 (100 μM) or PMA (10 nM).

### Western blot analysis

Diaphragm muscles from newborn and adult rat were dissected, frozen in liquid nitrogen, and stored at −80°C before use. The muscles were homogenized using a high-speed homogenizer (overhead stirrer, VWR International, Clarksburg, MD) in lysis buffer containing 150 mM NaCl, 20 mM Tris–HCl, pH 7.5, 2 mM EGTA, and 5 mM EDTA supplemented with 1% Triton X-100, 1 mM PMSF, 50 mM NaF, and 1 mM sodium orthovanadate from Sigma, (St. Louis, MO) and protease inhibitor cocktail (Sigma-Aldrich Corp., Saint Louis, MO, USA). Insoluble material was removed by centrifugation at 1000 g for 10 minutes. The supernatants were collected and centrifuged at 15000 g for 20 minutes. Finally, the resulting supernatants (total protein lysates) were collected. Protein concentrations were determined by using the Bio-Rad DC protein assay (Bio-Rad, Hercules, CA). Experimental procedures were performed to determine the linear and quantitative dynamic range for each target protein and the appropriate dilutions of samples were used for accurate and normalized quantitation by means of densitometric analysis. Protein samples of 15 or 30 μg were separated by 8% SDS-polyacrylamide electrophoresis and electrotransferred to PVDF membranes (Hybond™-P; Amersham, GE Healthcare). The membranes were blocked in Tris-buffered saline-0.1% Tween-20 (TBS-T) containing 5% (W/V) nonfat dry milk or in a blocking reagent to preserve phosphoprotein antigens (PhosphoBLOCKER™; Cell Biolabs, Inc.) and probed with the primary antibody overnight at 4°C. The membranes were then incubated with the secondary antibody and visualized enhanced chemiluminescence with the ECL kit (Amersham Life Science, Arlington Heights, IL).

In treated and/or stimulated muscles, the synaptic membranes were obtained. Synaptic and extrasynaptic parts of the diaphragm muscle were separated as previously described [25]. We performed control experiments to check that our protocol for dividing the diaphragm muscle into synaptic and extrasynaptic region was accurate. In some muscles, we repeated the process of separation and detected NMJs with TRITC-conjugated α-BTX. We also stained the nerves with an antibody against anti-neurofilament-200 and did not detect any nerves in extrasynaptic regions. The muscles were homogenized using a high-speed homogenizer (overhead stirrer, VWR International, Clarksburg, MD) in lysis buffer (see above) and the insoluble material was removed in the same way (by centrifugation at 1000 g for 10 minutes) but now the resulting supernatant was collected and centrifuged at 130000 g for 1 hour. The supernatant was the cytosolic fraction, and the pellet was the membrane fraction. To assess the separation of the membrane fraction from the cytosol, we used a goat antibody directed against Glyceraldehyde 3-phosphate dehydrogenase (GAPDH), a protein specific to the cytosolic fraction. GADPH immunoreactivity was not observed in any case in the membrane fraction. The samples were processed the same way as another sample of total protein (see below).

The blots were visualized with a VersaDoc 3000 (Bio-Rad, Hercules, CA). The densitometry of different bands was analyzed with the MetaMorph software. The integrated optical density of the bands was normalized to the background values and by actin protein. Also, as another loading control we used a total protein analysis (Sypro Ruby protein blot Stain, Bio Rad) to measure the total protein transferred on polyvininylidene difluoride (PVDF) membranes. In all the cases, the quantitative results obtained by using actin or total protein analysis were no different. The relative variations between the bands in the experimental samples and the control samples were calculated in the same image. The data were taken from densitometry measurements made in at least five separate experiments, plotted against controls. Data are mean values ± SD. Differences between groups were tested using the *t Student* test or *U* test (Mann–Whitney), and the normality of the distributions was tested with the Kolmogorov–Smirnov test. The criterion for statistical significance was *p* < 0.05 versus the control.

### Immunohistochemistry and confocal microscopy

Whole muscle mounts were processed by immunohistochemistry to detect the localization of the nPKCε isoform at the NMJ. LAL and diaphragm muscles from young adult rats were fixed with 4% paraformaldehyde for 30 minutes. After fixation, the muscles were rinsed with PBS and incubated in 0.1 M glycine in PBS. The muscles were permeabilized with 0.5% Triton X-100 in PBS, and nonspecific binding was blocked with 4% bovine serum albumin (BSA). Then, muscles were incubated overnight at 4°C in mixtures of three primary antibodies raised in different species (anti-nPKCε isoform antibody and anti-syntaxin and anti-neurofilament or syntaxin or anti-S100) and then rinsed. The muscles were then incubated for four hours at room temperature in a mixture of appropriate secondary antibodies. The AChRs were detected with α-BTX conjugated with TRITC. At least three muscles were used as negative controls as described above. For a better analysis of the localization of the nPKCε isoform at the NMJ, some muscles were processed to obtain semithin cross-sections from whole-mount multiple-immunofluorescent stained muscles. This method provided a simple and sensitive procedure for analyzing the cellular distribution of molecules at the NMJ [24].

Labeled NMJs from the whole-mount muscles and the semithin cross-sections were viewed with a laser-scanning confocal microscope (Nikon TE2000-E). Special consideration was given to the possible contamination of one channel by another. In experiments involving negative controls, the photomultiplier tube gains and black levels were identical to those used for a labeled preparation made in parallel with the control preparations. At least 25 endplates per muscle were observed, and at least six muscles were studied. Images were assembled using Adobe PhotoShop software (Adobe Systems, San Jose, CA) and neither the contrast nor brightness were modified.

### Electrophysiology

Diaphragm muscles from adult rats were removed surgically and incubated in a Sylgard-Petri dish containing normal Ringer solution (in mM) – NaCl 135, KCl 5, CaCl_2_ 2.5, MgSO_4_ 1, NaH_2_PO_4_ 1, NaHCO_3_ 15, glucose 11 – which was bubbled continuously with 95% O_2_, 5% CO_2_. Temperature and humidity were regulated at 26°C and 50%, respectively. Spontaneous miniature endplate potentials (MEPPs) and evoked endplate potentials (EPPs) were recorded intracellularly with conventional glass microelectrodes filled with 3 M KCl (resistance: 20–40 MW). Recording electrodes were connected to an amplifier (Tecktronics, AMS02), and a distant Ag-AgCl electrode connected to the bath solution via an agar bridge (agar 3.5% in 137 mM NaCl) was used as a reference. The signals were digitized (DIGIDATA 1322A Interface, Axon Instruments Inc, CA, USA), stored and computer-analyzed. The software Axoscope 9.0 (Axon Instruments Inc, CA, USA) was used for data acquisition and analysis. To prevent muscle contraction during EPP recordings, we used μ-conotoxin GIIIB (μ-CgTx-GIIIB, 3 μM −1.5 μM, from ICS, International Clinical Service GmbH, München). After a muscle fiber had been impaled, the phrenic nerve was continuously stimulated (70 stimuli at 0.5 Hz) using two platinum electrodes that were coupled to a pulse generator (CIBERTEC CS-20) linked to a stimulus isolation unit. The last 50 EPPs were recorded. We selected fibers with membrane potentials of no less than -70 mV and used only those results from preparations which did not deviate by more than 5 mV during the recording. The mean amplitude (mV) per fiber was calculated and corrected for non-linear summation (EPPs were usually more than 4 mV) [71] assuming a membrane potential of – 80 mV. Quantal content (M) was estimated by the direct method, which consists of recording mEPPs and EPPs simultaneously and then calculating the ratio: M = Average Peak EPP/Average Peak mEPP. We studied a minimum of 15 fibers per muscle and usually a minimum of 5 muscles in each type of experiment. The data given in Results are mean values ± SEM. Only one hemidiaphragm was used from each animal for a given experiment. We used the two-tailed Welch’s t-test (for unpaired values and variances were not assumed to be equal). Differences were considered significant at P < 0.05 (*).

### Denervation

Extensor digitorum longus (EDL) denervation. Young adult rats (30–40 months of age, male) were anesthetized with ketamine/xylazine (K/X; 0.1 ml/10 g body weight intraperitoneal injection of a solution containing 10 mg/ml ketamine and 1 mg/ml xylazine). To isolate the sciatic nerve, a 0.5 cm excision was made on the exterior side of the leg at approximately 1 cm over the level of knee. The excision was made carefully avoiding tissue injury, the sciatic nerve was cut about 1 mm from the nerve’s entrance into the muscle and a 1 cm segment was excised. The wound was then closed. At the times desired (1–3 days), the rats were anesthetized with pentobarbital and the EDL muscle was excised.

## Abbreviations

ACh: Acetylcholine
LAL: Levator auris longus muscle
nAChR: Nicotinic acetylcholine receptor
NMJ: Neuromuscular junction
PKC: Protein Kinase C
nPKCε: Epsilon PKC
PMA: Phorbol 12-myristate 13-acetate
RACKs: Receptors for activated C-kinase
PS: Phosphatidylserine
DAG: Diacylglycerol
εV1-2: Isozyme-selective translocation inhibitor
α-BTX: α-bungarotoxin conjugated TRITC
TrkB: Tyrosine kinase receptor B
BDNF: Brain derived neurotrophic factor
EDL: Extensor digitorum longus muscle
PLCγ1: Phosphoinositide-specific phospholipase C γ1
PI(4,5)P_2_: Phosphatidylinositol 4,5-biphosphate
IP_3_: Inositol tris-phosphate
NT: Nerve terminal
PDK: 3-phosphoinositide dependent protein kinase
